# Practical Notes on Diagnosis and Treatment

**Published:** 1908-06-27

**Authors:** 


### Practical Notes on Diagnosis and Treatment.

**Mitral Stenosis.**

According to some writers, mitral stenosis discovered in patients in whom there is no history of rheumatism is probably of congenital origin.

**Drug-Taking.**

Half the world believes that the taking of drugs is all that is required for the medical art, and that for every ache and pain or other bodily trouble to which humanity is subject, a remedy may be found. Thus mere drug-giving for every ache or pain is a popular want, and if it is found that some medical men pronounce that this is the first and only thing to do, it is not remarkable that so many patients flock to them.—*Sir Samuel Wilks.*

**Calomel in High Blood-Pressure.**

Small doses of calomel—½ to 1 grain—given every night for a short time and repeated at intervals is of great use in reducing undue blood pressure.—*Dr. Herringham.*

**Duodenal Ulcer.**

When once a duodenal ulcer has given rise to hæmorrhage, whether this be shown by hæmatemesis or melæna, the bleeding, it may be taken for granted, will be repeated, and the recurrence of the bleeding may be severe or even fatal. My conviction is that a second hæmorrhage ought not to be waited for, but that operation ought to be undertaken as soon as the first bleeding has ceased.—*Mr. Mayo Robson.*

